# Working Women in Large Towns

**Published:** 1890-05-24

**Authors:** 


### Working Women in Large Towns.

### III.—FACTORY GIRLS.

Such a heterogeneous mass of workers is comprised in our next class—Factory and Workroom Hands—that the only justification for grouping them together is this: that they are all "indoor hands," which being interpreted means that they are outdoor, so far as their own homes are concerned, and that they all (without one exception) come under the Factory Acts. As for the trades in which they are engaged, their name is legion. They make our clothes, from the handsome sealskin jacket of the West-end lady to the "balloon" coats of East-end 'Arries, and the boots which they themselves wear; they wash our linen; they supply, or rather help to supply, us with buttons, umbrellas, jam, matches, biscuits, black-lead, and a host of other things too numerous even to name.

But let us hasten to distinguish, or our need of police protection may be even greater than that which Mr. Augustine Birrell whimsically declares will be his, if he dare to pronounce between the rival merits of Charlotte Brontë and Jane Austen. If even factory girls resent being spoken of as a compact whole, and demand to be divided into at least two classes, what would be the feelings of skilled tailoresses or needlewomen if they should chance to find themselves lumped together, if only for a few brief moments, with laundresses and jam-girls? We will lose no time, then, in sub-dividing this large class, after the fashion of the botanists, into orders, and perhaps again into genera. And to begin at the lowest end of the scale, let us consider the case of

The Factory Girl Proper.

To every Londoner she must be a familiar figure, with her loud laugh and rough, noisy manners, her white apron and gilt earrings, and the thick fringe coming down almost to her eyebrows, which, with a wisp of hair behind, is considered by some of her kind sufficient head-gear, even for her "Sunday get-up." Still, by her, as by so many Londoners of the female sex, a hat trimmed with a profusion of gorgeously-coloured ostrich feathers is generally coveted, and not unfrequently procured, and we are irresistibly reminded of that much-despoiled bird in more ways than one when we see the marvellous head-dresses of some of these otherwise bedraggled working girls, and observe that they, too, take thought for their heads alone, and leave other parts of their persons to fend for themselves.

The factory girl is very often on her feet all day, yet the evening finds her full of energy and spirits, ready for any "larks" that may offer themselves, whether in the shape of a ramble in the streets, a visit to a music-hall or penny gaff, or a drink in some low public-house, to which she is treated by some young man who may or may not be a stranger to her. She is wild and untamed to an alarming degree; her parents, if she lives at home, have no control over her, for she pays for her board and lodging (at the rate of five or six shillings per week), and so feels herself quite independent. Very often she will break away from such slender home-ties as remain, and rent a room for herself with two or three of her "mates" for companions. Some observers reckon her wages as from seven-and-six to eleven shillings a-week; others (who are probably considering the lowest grade of all) put it at from four to eight shillings; but, however this may be, it would seem that when wages are pretty good she would rather take a holiday or two than trouble herself to earn more than about ten shillings. True, she has her hard times, for trade is often slack, and many of her industries leave her entirely in the lurch for some weeks in the year. Then she no doubt suffers, and sometimes suffers acutely; but her troubles do not seem to bring her wisdom, any more than do those of the flower-girl. She tides through them somehow, and then the old round begins again.

Worthy of mention and of all praise is the fact that she has the strongest feeling of *esprit-de-corps*—witness the firmness with which the match girls stood together in their late strike, and the generous help which the more fortunate ones accorded to those who were specially "hard-up." Even this, however, has its unfortunate side; for so exclusive is she, that she can hardly be persuaded to mix with any other class of workers. If she enters the doors of Mr. John Shrimpton's excellent Homes for Working Girls, it is only by mistake, as it were, and for a very brief period; and the People's Palace knows her not, Sir Edmund Currie regretfully remarking that it is impossible to do anything for factory girls, unless you lay yourself out to get them, and them alone.

This, then, is the type of the lowest class of factory girls. They are to be found in jam and sweetmeat factories, washing and filling bottles and jars, packing raisins, cutting lozenges, peeling oranges, filling surprise packets, and at certain seasons of the year picking over fruit. Again, they make rope, matches, and a hundred other things, many of them working in tobacco factories, which is a trade that pays comparatively high wages, but is considered by many to be detrimental to the girls' health. In ginger-beer and firework factories, again, they often meet with accidents, such as having an eye blown out, so that their life may be a dangerous as well as a hard and hand-to-mouth one. Moreover, we cannot doubt that while the typical factory-girl is such as we have tried to describe, there are many of her kind whose life is a hard and bitter struggle with poverty and privation, caused by want of skill or slack times, and often aggravated by ill-health. Where wages are so small, those who get the lowest pay must of necessity be hard pressed; and when the girls have relatives depending on them, as sometimes happens, it must be difficult, if not impossible, to make both ends meet.

But the most serious question which we have to ask ourselves with regard to these low-class factory girls is not—How can their wages be raised? but—How can they themselves be raised? They are improvident, fond of drink, low in their standards of comfort and of morality; and yet—so far, at least, as the East-end is concerned—every one of these girls can marry—will marry, and, if she follows the example of her mother, will very likely bring into the world a family of twelve, or even seventeen. True, many of that goodly band may not live through infancy, for the child-mortality among the lowest East-end classes is enormous, but what of those who do? Can we expect them to become useful, industrious, provident, respectable citizens?

